# TGF-β Signaling Promotes Glioma Progression Through Stabilizing Sox9

**DOI:** 10.3389/fimmu.2020.592080

**Published:** 2021-02-03

**Authors:** Min Chao, Nan Liu, Zhichuan Sun, Yongli Jiang, Tongtong Jiang, Meng Xv, Lintao Jia, Yanyang Tu, Liang Wang

**Affiliations:** ^1^ Departments of Neurosurgery, Tangdu Hospital, Fourth Military Medical University, Xi’an, China; ^2^ Departments of Experimental Surgery, Tangdu Hospital, Fourth Military Medical University, Xi’an, China; ^3^ Departments of Neurology, Xijing Hospital, Fourth Military Medical University, Xi’an, China; ^4^ State Key Laboratory of Cancer Biology, Department of Biochemistry and Molecular Biology, Fourth Military Medical University, Xi’an, China

**Keywords:** glioma, Sox9, transforming growth factor-β, migration, invasion

## Abstract

Gliomas are brain and spinal cord malignancies characterized by high malignancy, high recurrence and poor prognosis, the underlying mechanisms of which remain largely elusive. Here, we found that the Sry-related high mobility group box (Sox) family transcription factor, Sox9, was upregulated and correlated with poor prognosis of clinical gliomas. Sox9 promotes migration and invasion of glioma cells and *in vivo* development of xenograft tumors from inoculated glioma cells. Sox9 functions downstream of the transforming growth factor-β (TGF-β) pathway, in which TGF-β signaling prevent proteasomal degradation of the Sox9 protein in glioma cells. These findings provide novel insight into the wide interplay between TGF-β signaling and oncogenic transcription factors, and have implications for targeted therapy and prognostic assessment of gliomas.

## Introduction

Glioma is the most common primary central nervous system (CNS) malignant tumor, accounting for about 35–40% of intracranial tumors. Glioma is characterized by high rates of occurrence, invasiveness, and recurrence, with an extremely short overall survival time (OS) and high 5-year mortality rate (1). While the mechanisms underlying their pathogenesis remain largely elusive, gliomas, especially glioblastomas (GBM), often arise from aberrant differentiation of neural cells (2, 3). Genetic mutation is known to drive malignant transformation at least in part by “hijacking” neurodevelopmental programs (4, 5). Increasing evidence has suggested that Sox9, an indispensable transcription factor in the development of the nervous system, plays a pivotal role in the pathogenesis of glioma (6–8).

As a member of the Sry-related high mobility group box (Sox) transcription factors, Sox9 plays various important roles in the development of cartilage, sex organs, and the CNS (9, 10); Sox9 is also crucially involved in the self-renewal and differentiation of neural stem cells (NSCs) (6, 11). Consistent with its critical role in glial differentiation, Sox9 deregulation is closely related to the occurrence and development of glioma. We and others have demonstrated previously that Sox9, which is upregulated *via* various mechanisms, contributes to the occurrence and progression of glioma (12, 13).

Transforming growth factor-β (TGF-β) signaling, a canonical pathway regulating oncogenesis and tissue homeostasis, has been documented to participate in the pathogenesis of divergent malignancies including glioma (14–16). In particular, TGF-β pathway is a key regulator of glioma stem cells (GSCs). Shinojima et al. reported that TGF-β mediates homing of bone marrow-derived human mesenchymal stem cells (BM-hMSCs) to GSCs (17). Bruna et al. found that high TGF-β/Smad activity confers poor prognosis in glioma patients and promotes cell proliferation *via* platelet-derived growth factor B (PDGF-B) (18). Nonetheless, it remains to be fully understood how TGF-β signaling drives the progression of glioma especially considering the divergent genetic context of these clinical malignancies.

In the present study, we investigated the role of Sox9 in regulation of the malignant phenotypes of glioma cells, and explored upstream pathways responsible for Sox9 deregulation. We established that Sox9 overexpression underlies glioma pathogenesis, and TGF-β pathway plays an essential role in upregulating Sox9 and thereby promoting glioma progression.

## Materials and Methods

### Cells and Human Tissue Samples

U87, U373, and U251 cells were purchased from the Chinese Academy of Sciences Cell Bank in 2018. All cell lines were grown in high glucose Dulbecco’s Modified Eagle Medium (DMEM) (Hyclone, USA) supplemented with 10% FBS (Gemini, A49F74G) as well as 100 IU/ml penicillin and 100 μg/ml streptomycin (Hyclone, USA), and incubated in 5% CO_2_ at 37°C. Eighty-six cases of human gliomas were collected from the neurosurgical specimens of Tangdu Hospital, Fourth Military Medical University, China. All patients were operated for the first time, and all of them were confirmed to be glioma by pathological assays. Normal brain tissues of 14 patients who have encountered with traumatic brain injuries. The study was approved by the Research Ethics Committee of Tangdu Hospital of Fourth Military Medical University, China. All patients involved in this study have signed the informed consent before, and all specimens were handled anonymous processing according to ethical and legal standards. U251, U373, and U87 cell lines, before the western blot, CCK8, wound-healing, transwell, and animal experiments, were treated with TGF-β1 cytokines (Novoprotein, CA59) at the concentration of 5 ng/ml for 2 h, 20 min, and 1 h, respectively. U251 and U373 cell lines, before the western blot, CCK8, wound-healing, and transwell assays, were treated with an inhibitor of TGF-β receptors I/II (Selleck, LY2109761) at the concentration of 5 μM for 12 h. Cells were pretreated with TGF-β receptor inhibitors LY2109761 (Selleck), or DMSO control for 12 h before transfected with Sox9 overexpression. Cells were pretreated with proteasomal inhibitor MG132 (MCE) at the concentration of 25 μg/ml for 6 h. U251 cells were treated with 50 μg/ml of cyclohexamide (Sigma) or DMSO control for 1 h, then treated with TGF-β1 or vehicle control (19).

### Gene Knockdown *via* Vector-Based shRNAs

Stable gene knockdown in U251, U373, and U87 cell lines were achieved by infection with recombinant shRNA-expressing lentiviruses and subsequent selection with puromycin at a concentration of 5 μg/ml for about 2 weeks. The shRNA target sequences are as follows: NC, TTCTCCGAACGTGTCACGT; Sox9, GCATCCTTCAA TTTCTGTATA.

### CCK8 Assay

Cells were plated into a 96-well plate, and cultured at 37˚C with 5% CO_2_ for 12, 24, 48, and 72 h. There are five repeats of each sample. Subsequently, 10 μl CCK8 (5 mg/ml; Life Technologies) was added into 90 μl DMEM (10%FBS). The mixture was transferred into every sample and incubated at 37˚C with 5% CO_2_ for 2 h, the cells with CCK8 was detected by determining the optical density (OD) at 450 nm (Thermo Fisher Scientific, Waltham, MA, USA).

### Wound Healing Assays

U251 and U373 cells were seeded in six-well plates and cultured 24 h. A wound was then created by manually scraping the cell monolayer with a 200 μl pipette tip. The cultures were washed twice with PBS (Hyclone, USA) to remove floating cells. The cells were then incubated in serum-free DMEM. Cell migration into the wound was observed at three preselected time points (0, 24, and 48 h) in three randomly selected microscopic fields for each condition and time point. Images were acquired with a Nikon DS-5M Camera System mounted on a phase-contrast Leitz microscope and were processed using Image J.

### Transwell Assays

Cells were suspended in 100 μl serum-free DMEM and seeded in the top chambers of 24-well transwell plates (Costar, USA) coated with 100 μl Matrigel (BD Biosciences, Franklin Lakes, NJ, USA). The bottom chambers of the transwell plates were filled with 600 μl DMEM containing 10% FBS. Cells were allowed to migrate for 48 h at 37°C. By the time, the cells which invaded to the bottom chambers were fixed in methyl alcohol, and cells in the top chambers were removed using a cotton swab. Then cells were stained with 0.1% crystal violet. The fixed and stained cells were counted in five independent fields under a light microscope. At least three chambers were counted for each experiment. For the migration assays, a similar protocol was followed except for the replacement of the top chamber of the transwell plate with an uncoated chamber. The culture medium in the bottom chamber was replaced with DMEM containing 10% FBS, and cells were allowed to migrate for 24 h.

### Immunohistochemistry Assay

For immunohistochemistry (IHC), 8 μm sections of formalin-fixed and paraffin-embedded brain tissues were first de-waxed and rehydrated before antigen retrieval. The TGF-β1-antibody (1:100 dilution; Proteintech, China) and Sox9-antibody (1:250 dilution; Abcam, ab76997) were used for this study. After incubation with the primary antibodies, the tissues were rinsed and incubated for 1 h with Biotin-labeled secondary antibodies at room temperature (Molecular Probes 1:800). Nuclei were stained by Hematoxylin. Stained sections were examined under a light microscope and the positive cells in five high power fields (1 × 400) were counted for statistic study. The relative expression of TGF-β1 and Sox9 was analyzed by Graphpad *via* Spearman rank correlation test.

### Western Blotting

The five cases of peritumor brain tissues and glioma tissues from patients were collected from the neurosurgical specimens of Tangdu Hospital. Peritumor brain tissues were dissected 0.5–1.0 cm away from glioma core regions, which were further histologically confirmed by H&E staining. The study was approved by the Research Ethics Committee of Tangdu Hospital of Fourth Military Medical University, China. All patients involved in this study have signed the informed consent before, and all specimens were handled anonymous processing according to ethical and legal standards. The glioma and peritumor tissues, and total cell lysates were dissolved in middle RIPA Lysis buffer (Beyotime, China) with complete protease inhibitor cocktail (Roche, USA). The protein concentrations were determined by a protein assay kit (Beyotime, China). Twenty micrograms protein was separated with 12% sodium dodecyl sulfate-polyacrylamide gel electrophoresis (SDS-PAGE), and transferred onto a polyvinylidene difluoride membrane (Roche, USA), which was incubated with TBST containing 5% skim milk 2 h at room temperature; and then with rabbit anti-Sox9 (1:1,000, Abcam, ab185230), rabbit anti-β-actin (1:100,000, Abclonal, China) monoclonal antibodies overnight at 4°C and then with goat anti-rabbit monoclonal IgG (1:10,000; Abclonal, China) secondary antibodies at room temperature for 2 h, followed by chemiluminescence for visualization with an ECL kit (Genshare biological, China).

### Animal Experiments

All animal experiments were approved by the Ethics Committee of the Fourth Military Medical University, China. Nude immunocompromised mice were purchased from Fourth Military Medical University, Shanxi, China, and breeding colonies were maintained in SPF conditions. Xenografted transplantation of glioma cells into nude immunocompromised mice was performed as previously described. There are Sox9-NC group and Sox9-KD group for the U87 cell lines. After pre-transplant preparation of the recipient mice and anesthesia with 10% chloral hydrate. Isolated U87 cells of every group (10^7^ in 1 ml PBS) and were implanted into the under left axilla of nude mice by subcutaneous injection to establish the xenograft model. The weight change of each animal was measured twice a week. Tumor volumes were determined by measuring the length (a) and the width (b). The tumor volume (V) was calculated according to the formula V = ab^2^/2.

U87 glioma cells, which were infected by Sox9-NC and Sox9-KD respectively with GFP, were orthotopically implanted in nude mice. U87 cells were pretreated with TGF-β1 (5 ng/ml). Implantation of U87 cells into the brains of nude mice was performed under anesthesia. All procedures re-quiring anesthesia were performed using Chloral hydrate at the concentration of 10% (0.04 ml/10g) i.p. 3 μl of tumor cell suspension (10^5^ cells/μl) was stereotactically inoculated in the right forebrain using a 5 μl syringe. On day 21, mice were anesthetized, and the brains were removed under perfusion with sterile 0.9% NaCl and paraformaldehyde. The brains were fixed in paraformaldehyde for 6 h and dehydrated in 10, 20, and 30% sucrose. Brain tissues were Frozen sections of brain tissues were prepared and the fluorescence was detected with the laser confocal microscope. The tissues were confirmed by H&E staining.

### Statistical Analysis

Independent samples were analyzed by using two-sided independent Student’s t-tests with Graphpad 7.0. Relative expression of TGF-β1 and Sox9 was analyzed *via* Spearman rank correlation test with Graphpad 7.0. Image J was used to cell counts, measurement of migrated distance, relative quantitation of western blot. All statistical results from the quantitative analysis of the *in vitro* experiments are presented as means ± SEM. *p* values < 0.05 were considered statistically significant.

## Results

### Sry-Related High Mobility Group Box 9 Expression Correlates With Progression of Clinical Glioma

**Figure 1:**
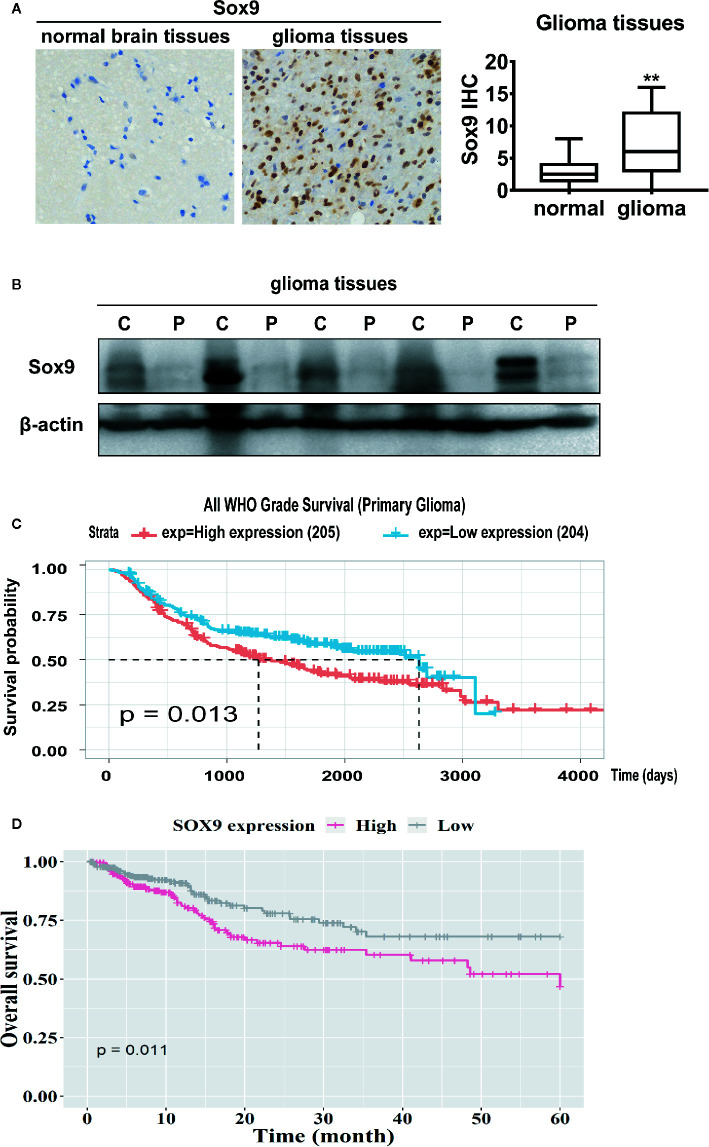
Immunohistological stain of Sox9 in 14 normal brain tissues and 86 glioma tissues (*p* < 0.01). **(A)** Western blot of Sox9 in glioma tissues (C) and paired adjacent tissues (P) of five patients. **(B)** Analysis of the expression of Sox9 and the prognosis of glioma patients from CGGA database **(C)** and TCGA database **(D)**. Value of p < 0.01 (**) was considered statistically significant.

Sox9 has been documented as an oncogenic transcription factor in various malignancies (20, 21). We examined the expression of Sox9 in clinical glioma. Immunohistochemical staining and western blot of five patients showed that Sox9 was upregulated in glioma tissues when compared with the peritumor tissues (**Figures 1A, B**). Consistently, data from Chinese Glioma Genome Atlas (CGGA) and the Cancer Genome Atlas (TCGA) suggested that high Sox9 expression correlates with short survival of glioma patients (**Figures 1C, D**). Thus, Sox9 is a predictive biomarker for the pathogenesis and prognosis of clinical glioma.

### Sry-Related High Mobility Group Box 9 Promotes Malignant Phenotypes of Glioma Cells

**Figure 2:**
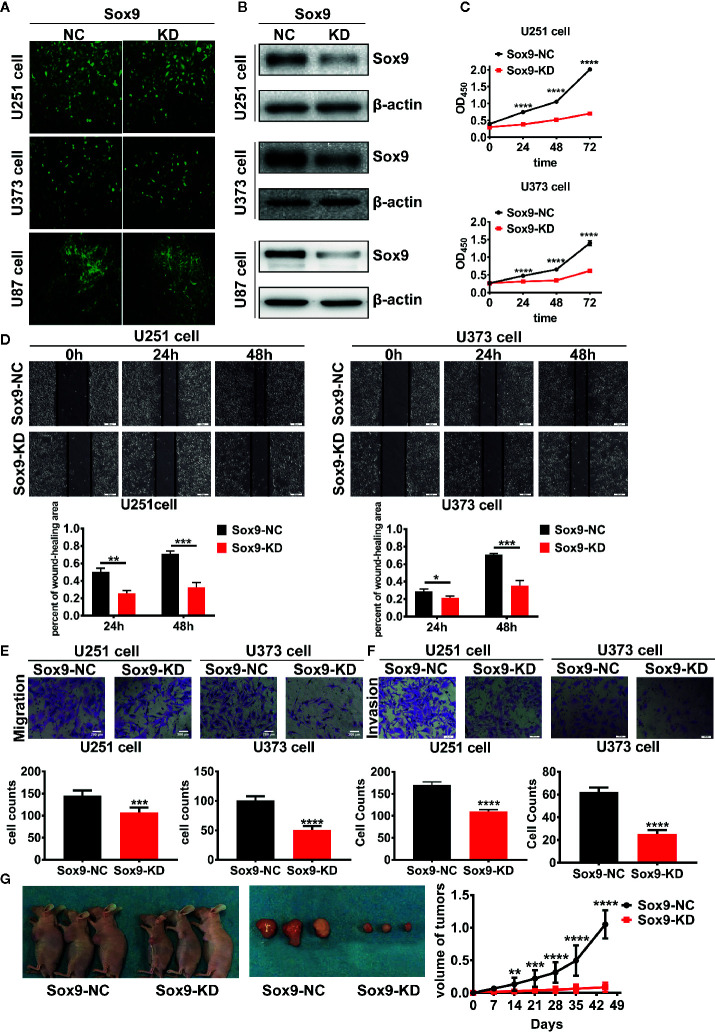
The transfected efficient of Sox9-KD lentivirus in U251, U373, and U87 cells were detected by GFP stain. **(A)** U251, U373, and U87 Sox9-kd stable cell lines were detected by Western blot. **(B)** CCK8 assay of both U251 Sox9-kd cells and U251 Sox9-nc cells, as well as U373 Sox9-kd cells and U373 Sox9-nc cells in 24, 48, and 72 h (n = 3, *p* < 0.001). **(C)** Wound healing assay of both U251 Sox9-kd cells and U251 Sox9-nc cells at 0, 24 (*p* < 0.01), and 48 h (*p* < 0.005), as well as U373 Sox9-kd cells and U373 Sox9-nc cells (24 h: *p* < 0.05; 48 h: *p* < 0.005). **(D)** Transwell (migration) assay in U251 Sox9-kd cells and U251 Sox9-nc cells (*p* < 0.005), as well as in U373 Sox9-kd cells and U373 Sox9-nc cells (*p* < 0.001). **(E)** Transwell (invasion) assay in U251 Sox9-kd cells and U251 Sox9-nc cells (*p* < 0.001), as well as in U373 Sox9-kd cells and U373 Sox9-nc cells (*p* < 0.001). **(F)** Tumorigenesis xenografts with U87 Sox9-nc cells and U87 Sox9- kd cells after 40 days (n = 10). **(G)** Statistical analysis was performed using a two-tailed independent t-test. Values of *p* < 0.05 (*), *p* < 0.01 (**), *p* < 0.005 (***), and *p* < 0.001 (****) were considered statistically significant.

To determine the biological function of Sox9 in glioma cells, Sox9 was knocked down in glioma cell lines, U251, U373, and U87, *via* shRNAs expressed from recombinant lentiviral vectors (**Figures 2A, B**). CCK8 assays indicated that Sox9 silencing resulted in a moderate growth inhibition of glioma cells (**Figure 2C**). Knockdown of Sox9 also remarkably reduced the migration capability of U251 and U373 cells as shown in wound-healing (**Figure 2D**) and Transwell (**Figure 2E**) assays. Similarly, Sox9 knockdown significantly decreased the invasiveness of glioma cells in a Transwell assay (**Figure 2F**). Sox9 downregulation also inhibited the development of xenograft tumors in nude mice challenged with the U87 glioma cells (**Figure 2G**). Thus, Sox9 plays an essential role in maintenance of the malignant phenotypes of glioma cells.

### Sry-Related High Mobility Group Box 9 Functions Downstream of Transforming Growth Factor-β Signaling to Promote Glioma Pathogenesis

**Figure 3:**
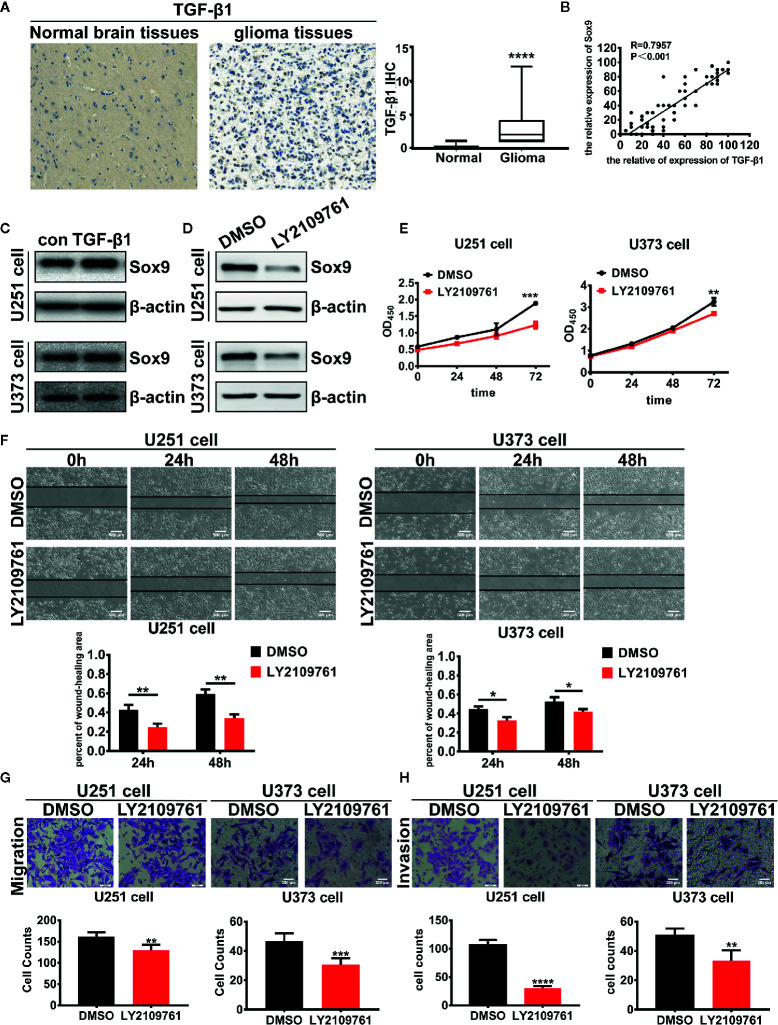
Expression of TGF-β1 in 86 glioma tissues and 14 normal brain tissues were detected by immunohistochemistry (*p* < 0.001). **(A)** Analysis of the correlation of TGF-β1 and Sox9 in IHC assays (R = 0.7957). **(B)** Expression of Sox9 was detected by western blot after that U251 cells were treated with TGF-β1 (5 ng/ml) at 2 h and U373 cells were treated at 20 min. **(C)** Expression of Sox9 was detected by western blot after that U251 cells and U373 cells were treated with LY2109761 (5 μM) at 12 h. **(D)** CCK8 assay of both DMSO-treated U251 cells and LY2109761-treated U251 cells (*p* < 0.005), as well as in U373 cells (*p* < 0.001). **(E)** Wound-healing assay of both DMSO-treated U251 cells and LY2109761-treated U251 cells at 0 h, 24 h (*p* < 0.01), and 48 h (*p* < 0.01), as well as in U373 cells (24 h: *p* < 0.05; 48 h: *p* < 0.05). **(F)** Transwell (migration) assay in DMSO-treated U251 cells and LY2109761-treated U251 cells (*p* < 0.01), as well as in U373 cells (*p* < 0.001). **(G)** Transwell (invasion) assay in DMSO-treated U251 cells and LY2109761-treated U251 cells (*p* < 0.001), as well as in U373 cells (*p* < 0.01). **(H)** Statistical analysis was performed using a two-tailed independent t-test. Values of *p* < 0.05 (*), *p* < 0.01 (**), *p* < 0.005 (***), and *p* < 0.001 (****) were considered statistically significant.

**Figure 4:**
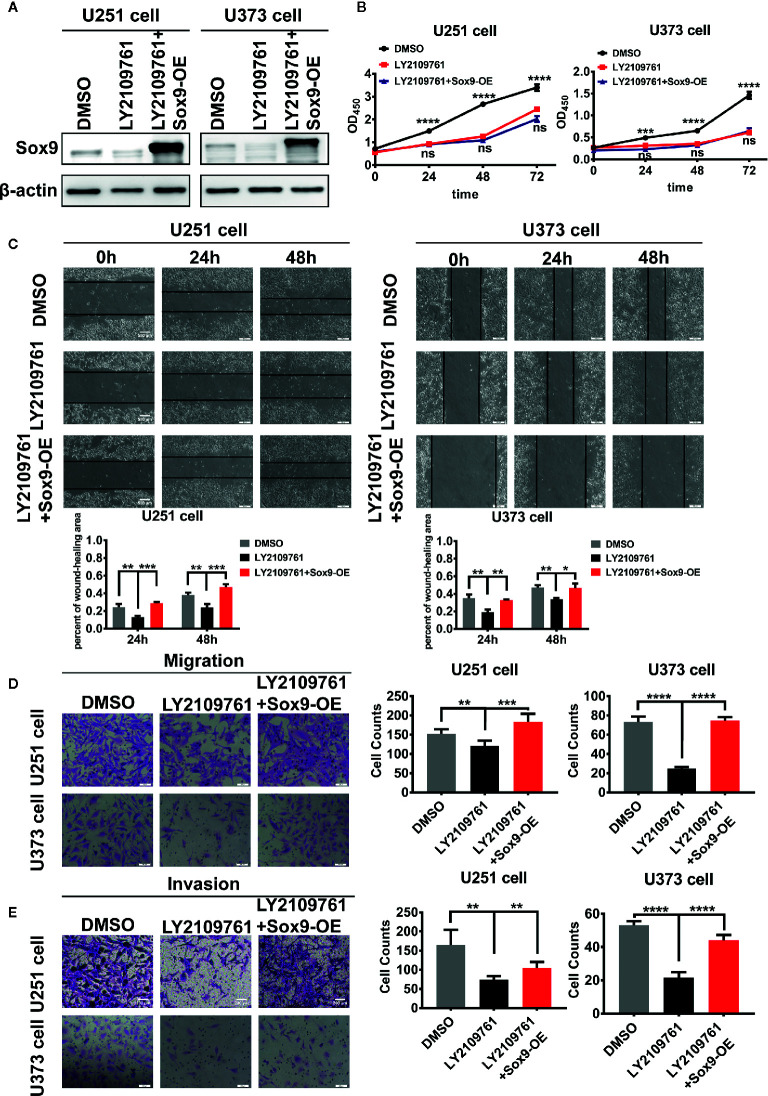
Expression of Sox9 was detected by western blot after that U251 cells and U373 cells were treated with LY2109761 (5 μM) at 12 h, and then while Sox9 was overexpressed (OE). **(A)** CCK8 assay among DMSO-treated U251 cells, LY2109761-treated U251 cells, and LY2109761+Sox9-OE-treated U251 cells, as well as in U373 cells (LY2109763 *vs* LY2109763+Sox9-OE: ns). **(B)** Wound-healing assay of DMSO-treated U251 cells, LY2109761-treated U251 cells, and LY2109761+Sox9-OE-treated U251 cells at 0, 24 (LY2109763 *vs* LY2109763+Sox9-OE: *p* < 0.005), and 48 h (LY2109763 *vs* LY2109763+Sox9-OE: *p* < 0.005), as well as U373 cells (LY2109763 *vs* LY2109763+Sox9-OE: 24 h: *p* < 0.0.01; 48 h: *p* < 0.05). **(C)** Transwell (migration) assay in among DMSO-treated U251 cells, LY2109761-treated U251 cells, and LY2109761+Sox9-OE-treated U251 cells (LY2109763 *vs* LY2109763+Sox9-OE: *p* < 0.005), as well as in U373 cells (LY2109763 *vs* LY2109763+Sox9-OE: *p* < 0.001). **(D)** Transwell (invasion) assay in among DMSO-treated U251 cells, LY2109761-treated U251 cells, and LY2109761+Sox9-OE-treated U251 cells (LY2109763 *vs* LY2109763+Sox9-OE: *p* < 0.005), as well as in U373 cells (LY2109763 *vs* LY2109763+Sox9-OE: *p* < 0.001). **(E)** Statistical analysis was performed using a two-tailed independent t-test. Values of *p* < 0.05 (*), *p* < 0.01 (**), *p* < 0.005 (***), and *p* < 0.001 (****) were considered statistically significant.

We next investigated the oncogenic signal pathways responsible for Sox9 upregulation in glioma cells. TGF-β signaling has been established to promote the progression of various cancers including glioma through substantially affecting the profiles of gene expression in neoplastic cells (22), which is reminiscent the role of oncogenic transcription factors (**Figure 3A**). The expression of Sox9 was positive correlated with TGF-β1 *via* the analysis of TGF-β1 and Sox9 in IHC of glioma tissues (**Figure 3B**). In line with these reports, we found that Sox9 was upregulated by treatment of glioma cells with recombinant TGF-β1 (**Figure 3C**), and Sox9 levels decreased when TGF-β signaling was blocked by a selective inhibitor, LY2109761 (**Figure 3D**). We have clarified that the migration and invasion of glioma cells, treated with recombinant TGF-β1, were significantly increased (23). And inhibition of TGF-β pathway caused remarkably reduced cell proliferation, migration, and invasion in CCK8, wound-healing, and transwell assays (**Figures 3E–H**). However, further overexpression of Sox9 in these cells rescued the capability of migration and invasion (**Figures 4A, C, D, E**), but not the ability of proliferation (**Figure 4B**). Next, to further confirm the relationship between TGF-β and Sox9, we implanted the U87 cells orthotopically to establish xenografts. As we can see, tumors, treated with TGF-β1, were more aggressive. The proliferation of Sox9-NC group and Sox9-KD group, treated with TGF-β1, showed non-significance (which were consistent with the results *in vitro*). The intracranial tumors of Sox9-NC group were more invasive, while the tumors of Sox9-KD groups were limited (**Figure S1A**). These data suggest that Sox9 is a functional target of TGF-β signaling in promoting glioma pathogenesis.

### Transforming Growth Factor-β Signaling Represses Proteasomal Degradation of Sry-Related High Mobility Group Box 9 in Glioma Cells

**Figure 5:**
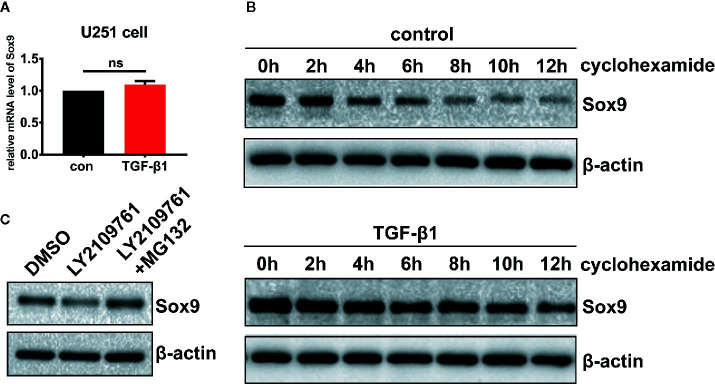
Glioma cells treated with TGF-β1 (5 ng/ml) at 2 h, qRT-PCR showed that the expression of Sox9 mRNA had no changes. **(A)** Treatment of glioma cells with CHX, western blot displayed that TGF-β could stabilize the expression of Sox9 protein. **(B)** Glioma cells treated with LY2109761 showed decrease of Sox9 protein, and MG132 could counteract the effect of LY2109761 to Sox9 protein **(C)**.

The mechanisms underlying Sox9 regulation by TGF-β pathway in glioma cells were probed. Inhibition of TGF-β signaling decreased the level of Sox9 protein (**Figure 3C**), but not that of the mRNA (**Figure 5A**). Treatment of glioma cells with cycloheximide (CHX), which prevents translocation of elongating ribosomes, revealed that TGF-β pathway protected Sox9 protein from the degradation (**Figure 5B**). In addition, the proteasome inhibitor MG132 counteracted the decrease in Sox9 protein levels induced by the inhibitor of TGF-β signaling (**Figure 5C**). These results suggest that TGF-β signaling represses proteasomal degradation of Sox9 in glioma cells.

## Discussion

TGF-β pathway has been well documented to expedite the pathogenesis and recurrence of gliomas by extensively affecting the gene expression profiles of transforming or malignant cells (18, 24). While TGF-β acts *via* the specific heterodimer receptors, TGFBRI/II, to phosphorylate the Smad family proteins, which is subsequently imported into the nucleus and regulate target gene expression (25–27). Alternatively, TGF-β activates Ras/MAPK pathway *via* Smad-independent signaling to orchestrate gene expression and cell behaviors (28, 29). However, little is known how TGF-β signaling regulates transcription factors other than the Smad proteins in the context of glioma cells. We found here that Sox9, a transcription factor commonly overexpressed in various glioma and glioblastoma, is upregulated by TGF-β signaling. Consistent with previous reports in chondrocytes (19, 30), we established that the regulation occurs in the posttranslational level, i.e. TGF-β impairs the degradation of the Sox9 protein. Further study in glioma cells revealed that TGF-β signaling reduces the proteasomal degradation of Sox9. Sox9 is a critical regulatory target of TGF-β since its overexpression rescued the malignant phenotypes of glioma cells caused by inhibition of TGF-β signaling. Nonetheless, additional investigations are needed to determine how TGF-β signaling reduces the proteasmal degradation of Sox9. Sox9 has been reported as targets for proteasomal degradation after ubiquitination by the E3 ligase FBW7 or UBE3A (31, 32). We will further explore that whether TGF-β decreases the ubiquition of Sox9 *via* FBW7 or UBE3A. Carcinogenesis in many tissues has been found to revive a transcriptional network involved in embryonic development. In the central nervous system, Sox9 plays an important role in the differentiation of cranial neural crest cells (8), and was reported as an astrocyte-specific nuclear marker in adult brain outside the neurogenic regions (33). We found here that Sox9 was overexpressed in clinical gliomas, and correlated with a poor prognosis of glioma patients. Sox9 knockdown resulted in significantly suppressed proliferation, migration, and invasion of glioma cells, as well as impaired *in vivo* tumor development in a xenograft model, suggesting that Sox9 facilitates the formation of primary tumors probably *via* improving local invasion. Thus, it is worth additional investigation whether Sox9 overexpression promotes cell transformation through ablating the orchestrated differentiation of neural stem cells or astrocyte progenitors. In terms of the molecular mechanisms downstream of Sox9, Liu et al. found that Sox9 can promote glioma metastasis *via* Wnt/β-Catenin pathway (34); Glasgow et al. demonstrated that Sox9 determines gliogenesis and tumorigenesis of gliomas through differentially regulating the gene of NFIA, which is attributed to different modes of long-range enhancer interaction (20). While it is largely unknown whether Sox9 participates in potential cross-talks with other cancer-driving pathways and whether Sox9 play distinct roles dependent upon the molecular subtypes of glioma, our study highlights the function of Sox9 as an oncogenic transcription factor, and has implications for targeted therapy and prognostic assessment of clinical gliomas.

## Data Availability Statement

The raw data supporting the conclusions of this article will be made available by the authors, without undue reservation.

## Ethics Statement

The studies involving human participants were reviewed and approved by the Research Ethics Committee of Tangdu Hospital of Fourth Military Medical University, China. The patients/participants provided their written informed consent to participate in this study. The animal study was reviewed and approved by the Ethics Committee of the Fourth Military Medical University, China.

## Author Contributions

MC and NL performed most experiments, analyzed data, and wrote the manuscript. ZS participated in the animal experiment. YJ and TJ participated in the cell culture assays. MX collected glioma tissue samples. LJ revised the article. YT and LW designed the overall study, supervised the experiments, analyzed the results, and wrote the paper. All authors contributed to the article and approved the submitted version.

## Funding

This study was supported by the following: 1. The National Natural Science Foundation of China, grant no. 81702458, Recipient: NL. 2. The National Natural Science Foundation of China, grant no. 81772661, Recipient: LW. 3. The National Natural Science Foundation of China, grant no. 81572983, Recipient: YT.

## Conflict of Interest

The authors declare that the research was conducted in the absence of any commercial or financial relationships that could be construed as a potential conflict of interest.
