# Supplementary material for: TGF-β Signaling Promotes Glioma Progression Through Stabilizing Sox9
Source: Front Immunol. 2021 Feb 3;11:592080. doi: 10.3389/fimmu.2020.592080 (PMC7886799; doi:10.3389/fimmu.2020.592080)
Supplement: Supplementary file 1 [file DataSheet_1.docx]

Supplementary Material

## Supplementary Figures

**
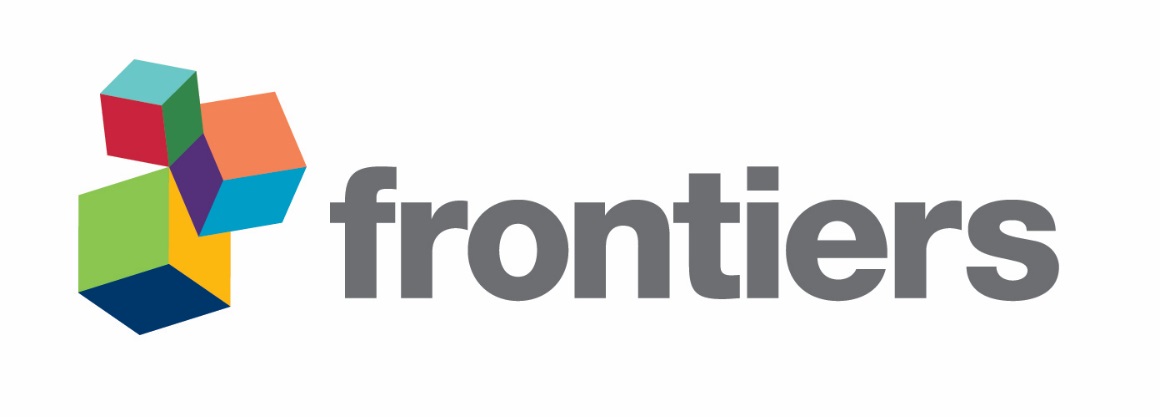
**
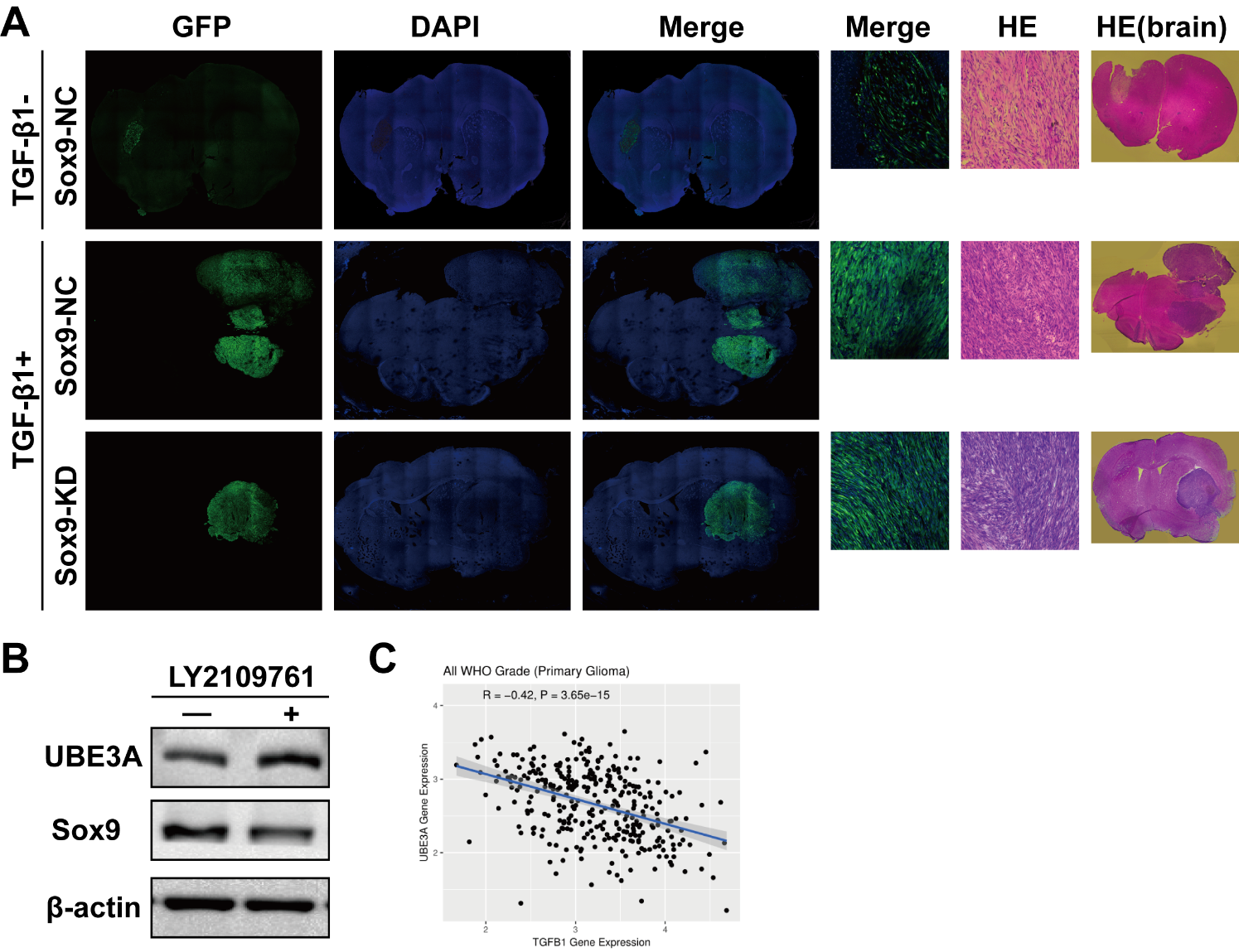


**Supplementary Figure 1.** Intracranial tumorigenesis in nude mice. (A) U251 cells treated with LY2109761 for 12h, expression of UBE3A and Sox9 were detected with Western Blot assays. (B) The CGGA database showed that UBE3A is negative correlation with the expression of TGF-β1. (C)

**
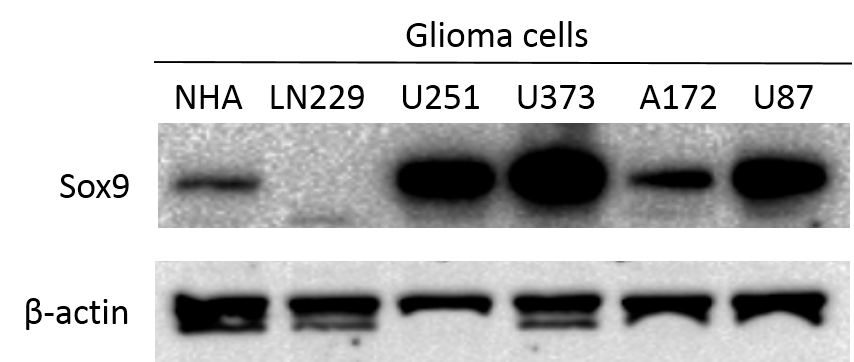
**

**Supplementary Figure 2.** Expression of Sox9 in glioma cells, Sox9 was highly expressed in U251, U373 and U87 cells.
